# Insights into the mechanism of isoenzyme-specific signal peptide peptidase-mediated translocation of heme oxygenase

**DOI:** 10.1371/journal.pone.0188344

**Published:** 2017-11-20

**Authors:** Bianca Schaefer, Kohji Moriishi, Soenke Behrends

**Affiliations:** 1 Department of Pharmacology, Toxicology and Clinical Pharmacy, University of Braunschweig—Institute of Technology, Braunschweig, Germany; 2 Department of Microbiology, Faculty of Medicine Yamanashi University, Yamanashi, Japan; Maastricht University, NETHERLANDS

## Abstract

It has recently been shown that signal peptide peptidase (SPP) can catalyze the intramembrane cleavage of heme oxygenase-1 (HO-1) that leads to translocation of HO-1 into the cytosol and nucleus. While there is consensus that translocated HO-1 promotes tumor progression and drug resistance, the physiological signals leading to SPP-mediated intramembrane cleavage of HO-1 and the specificity of the process remain unclear. In this study, we used co-immunoprecipitation and confocal laser scanning microscopy to investigate the translocation mechanism of HO-1 and its regulation by SPP. We show that HO-1 and the closely related HO-2 isoenzyme bind to SPP under normoxic conditions. Under hypoxic conditions SPP mediates intramembrane cleavage of HO-1, but not HO-2. In experiments with an inactive HO-1 mutant (H25A) we show that translocation is independent of the catalytic activity of HO-1. Studies with HO-1 / HO-2 chimeras indicate that the membrane anchor, the PEST-domain and the nuclear shuttle sequence of HO-1 are necessary for full cleavage and subsequent translocation under hypoxic conditions. In the presence of co-expressed exogenous SPP, the anchor and the PEST-domain are sufficient for translocation. Taken together, we identified the domains involved in HO-1 translocation and showed that SPP-mediated cleavage is isoform-specific and independent of HO-activity. A closer understanding of the translocation mechanism of HO-1 is of particular importance because nuclear HO-1 seems to lead to tumor progression and drug resistance.

## Introduction

Signal peptide peptidase (SPP) is a 42 kDa glycoprotein belonging to the family of aspartyl proteases [1]. SPP is located in the endoplasmic reticulum (ER) membrane and catalyzes intramembrane proteolysis of divergent substrate proteins in a number of very different physiological situations [1,2]. In contrast to many other proteases, no consensus cleavage site based on the amino acid sequence of the substrate has been reported. The classical function of SPP is the clearance of small peptide fragments from the ER membrane that derive from signal sequences after cleavage by signal peptidase [1,3]. Examples of such classical substrates are the signal peptides of prolactin or pro-calcitonin [4–6]. After cleavage by SPP within the ER membrane, fragments can be released into the cytosol or the ER lumen. In the ER lumen, these fragments attach to MHC class I proteins leading to antigen presentation on the cell surface [7]. The signal sequences of MHC class I proteins themselves are processed by SPP and subsequently bind to other processed MHC class I proteins in the ER lumen (HLA-E) [8]. Extracellular presentation of HLA-E protects non-infected cells from cytotoxic action by natural killer cells. While SPP thus supports a healthy response to infection, it can also be hijacked by viruses that use the protease to process viral protein [9–11]: For example, SPP cleaves the hepatitis core antigen and promotes the release of mature core from the ER membrane which is crucial for the production of infectious particles [12–14]. Based on this finding, SPP has been suggested as a novel drug target for chronic hepatitis C [15]. In a cell culture based proteomics screen using SPP-specific knock out cells, heme oxygenase-1 (HO-1), the rate limiting enzyme in the degradation of heme, was identified as a novel SPP substrate [16]. In the same year it was shown that SPP-mediated nuclear localization of HO-1 promotes cancer cell proliferation and invasion independent of its enzymatic activity [17].

Heme oxygenases are type II membrane proteins, which are anchored to the ER membrane with their hydrophobic carboxy-termini [18]. There are two isoforms in mammalians: inducible HO-1 (33 kDa) and constitutive HO-2 (36 kDa) [19,20]. The physiological function of heme oxygenase is heme degradation [21]. While HO-2 is constitutively expressed at the ER-membrane, HO-1 is induced by heme, infection and other stimuli and has the ability to translocate to the cytosol and nucleus [22,23,16,17]. Translocation of HO-1 can be mediated by hypoxia or hemin [23,22]. High levels of translocated HO-1 are detected in malignant cells and tumor tissue [17,24,25]. Furthermore, translocated HO-1 is suspected to contribute to resistance against the drugs imatinib in chronic myelogenous leukemia [26,27] and bortezomib in multiple myeloma [28].

In the current study, we used confocal laser-scanning microscopy and co-immunoprecipitation to investigate SPP-mediated HO translocation. Both isoforms of HO could be co-immunoprecipitated with endogenous SPP under conditions of normoxia. HO-1 was cleaved by SPP under conditions of hypoxia while HO-2 was highly resistant to SPP cleavage. Translocation studies with HO-1 mutants showed, that translocation is independent of HO activity, but dependent on the presence of the carboxy-terminus of HO-1. Experiments with HO-1 / HO-2 chimeras identified the sequences involved in translocation of HO-1 in the presence of overexpressed SPP or under conditions of hypoxia.

## Materials and methods

Unless stated otherwise, chemicals were purchased in highly purified quality from Sigma-Aldrich Chemie GmbH (Steinheim, Germany) or AppliChem GmbH (Darmstadt, Germany). Cell culture media were received from Life Technologies GmbH, Invitrogen™ (Darmstadt, Germany) or BIO & Sell e. K. (Feucht, Germany).

### Cloning of HO constructs, CPR, BVR and HA-SPP constructs

Wild type HOs, the anchorless construct GFP-HO-1-ΔC266, cytochrome P450 reductase (CPR) and biliverdin reductase (BVR) were cloned as described before [23,29]. Mutants were created using the QuickChange Lightning Site-Directed Mutagenesis Kit (Agilent Technologies Deutschland GmbH, Böblingen, Germany). To clone new constructs we used restriction enzymes from NEB according to manufacturer’s recommendations (New England Biolabs GmbH, Frankfurt/Main, Germany). To create the inactive HO-1 mutant we mutated histidine 25 into alanine as described by Ishikawa et al. 1992 [30]. We changed serine in position 275 to alanine and phenylalanine in position 276 to leucine as described in Hsu et al. [17] for cloning the HO-1 anchor mutant. The double fluorescence-tagged HO-1 construct was cloned by inserting HO-1-CFP into pEYFP-C1. HO-1-CFP was kindly provided by Dr. Esther Meyron-Holtz (Technion, Israel Institute of Technology, Haifa, Israel). For cloning of deletion mutants and chimeras we used artificial genes from Geneart^®^ (Life Technologies GmbH, Invitrogen™, Darmstadt, Germany) with silent mutations in the DNA sequence of HO. Deletion mutants were cloned with artificial gene 1 inserted into HO-1 *Kpn*I/*Bam*HI and subsequently cut *Bgl*II or *Bsr*GI. For the chimeras we made silent mutations to insert a *Bst*EII restriction site into HO-2 and an *Xma*I restriction site into the artificial gene 2. HO-1 / HO-2 chimeras were cloned by inserting the *Bsr*GI or *Xma*I cut artificial gene 2 into HO-2 *Bst*EII/*Sal*I or by inserting the artificial gene 2 *Bst*EII/*Eco*RI. The HO-1-HO-2 chimera was cloned by inserting artificial gene 3 *Apa*I/*Bam*HI into HO-1. For imaging and co-immunoprecipitation all constructs described above were cloned into vectors that allow fusion of the target gene with fluorescent proteins (pEXFP-C1 or pEXFP-N1, Clonetech, Heidelberg, Germany). Wild type HA-SPP and the catalytically inactive mutant HA-SPP mut (D219A) were kindly provided by Kohji Moriishi (Department of Microbiology, Faculty of Medicine Yamanashi University, Yamanashi, Japan). Mutations in SPPs PAL motif were generated as described above.

### Cell culture

HEK293 cells [31] (Leibniz Institute DSMZ, German Collection of Microorganisms and Cell Cultures, Braunschweig, Germany, DSMZ-No. ACC 305) were cultivated in Dulbecco’s Modified Eagle’s Medium High Glucose with 10% fetal bovine serum and 1% Penicillin/Streptomycin at 37°C with 5% CO_2_. For confocal laser scanning analyses Lipofectamine^®^ LTX & PLUS™ Reagent (Life Technologies GmbH, Invitrogen™, Darmstadt, Germany) was used for transfection according to manufacturer’s recommendations. The incubation time before imaging was at least two days. For Western blot analysis and co-immunoprecipitation cells were transfected with polyethylenimine (Polyscience, Inc., Warrington, USA). Before harvesting cell extracts they were cultured at least two more days under normoxia for precipitation with HA- or IgG control-antibody and three more days under normoxia or one more day under normoxia followed by 48 h hypoxia (1% O_2_) for precipitation with SPP- or IgG control antibody.

### Cell extracts

Cell extracts were made by sonification of scraped HEK293 cells (Sonoplus HD 2070, Bandelin electronic GmbH & Co. KG, Berlin, Germany) in triethanolamine-lysis-buffer (50 mM TEA, 1 mM EDTA, pH = 7.4) containing one tablet cOmplete protease inhibitor cocktail per 50 ml (Roche Diagnostics Deutschland GmbH, Mannheim, Germany) and subsequent centrifugation for 30 min at 21 000 x g and 4°C. For YFP-HO-1-CFP cell extracts were made as described by Schrul et al. [32]. Overall protein concentration of the cell extracts was determined by Bradford assay [33].

### Co-immunoprecipitation

Protein A Sepharose™ CL-4B beads (GE Healthcare Bio-Sciences AB, Uppsala, Sweden) were blocked overnight with BSA and milk. The next day cell extracts with the same amount of protein per sample (1.5-3 mg per experiment) were incubated for 2 h at 4°C with precipitation antibody (4–8 μg, rabbit-anti-HA, H6908, Sigma-Aldrich Chemie GmbH, Steinheim, Germany, AB_260070 / 4–6 μg, rabbit-anti-SPP, A304-404A, BIOMOL GmbH, Hamburg, Germany, AB_2617109, 2 μg rabbit-anti-IgG, 12–370, Merck KGaA, Darmstadt, Germany, AB_145841). Afterwards blocked beads were incubated with cell extracts and precipitation antibody for two more hours at 4°C. Subsequently the samples were centrifuged for 1 min at 300 x g and 4°C. The beads were washed three times with 1 ml of phosphate-buffered saline containing 1 tablet cOmplete protease inhibitor cocktail per 50 ml and finally eluted with 75 μl of SDS sample-buffer by 3 min cooking at 99°C. After bromophenol blue was added 35 μl of each eluate were analysed.

### SDS-PAGE and Western blot

For Western blot analysis cell extracts containing 90 μg protein extract and the equivalent volume of sodium dodecyl sulphate (SDS) sample-buffer (1% SDS, 100 mM DTT, 50 mM Tris, 30% Glycerol, pH = 7.5) were used. The samples for detection with anti-HA or anti-SPP were not cooked to keep the SPP homodimers at 95 kDa intact for detection [34]. All other samples were cooked for 3 min at 99°C. Afterwards bromophenol blue was added and the samples were loaded on 10% gels as the eluates from co-immunoprecipitation. PageRuler™ Prestained Protein Ladder and PageRuler™ Unstained Protein Ladder (Thermo Scientific, Walthram, USA) were used for size control. After SDS-PAGE gels were blotted on nitrocellulose membranes, stained with Ponceau S and blocked for at least 1 hour in TBST buffer (10 mM Tris–HCl, 150 mM NaCl, 0.1% Tween 20, pH = 8.0) containing 5% non-fat dry milk. For detection of the co-immunoprecipitates we used mouse-anti-GFP (1:1000, 11814460001, Sigma-Aldrich Chemie GmbH, Steinheim, Germany / Roche Diagnostics Deutschland GmbH, Mannheim, Germany, AB_390913) as primary antibody and horseradish peroxidase-conjugated anti-mouse-IgG as secondary antibody (1:2000, 7076S, Cell Signaling Technology, Inc., Danvers, USA, AB_10695470). The used mouse-anti-GFP-antibody is able to detect all used XFPs. For detection of SPP or SPP mutants in cell extracts we used rabbit-anti-HA (1:2000, H6908, Sigma-Aldrich Chemie GmbH, Steinheim, Germany, AB_260070) or rabbit-anti-SPP (1:2000, A304-404A, BIOMOL GmbH, Hamburg, Germany, AB_2617109) as primary antibody. For HO variants, BVR and CPR we used rabbit-anti-GFP (1:2000, 632592, Clontech Laboratories, Inc., Mountain View, USA, AB_2336883), which is able to detect all used XFPs. As secondary antibody for Western blots we used a horseradish peroxidase-conjugated anti-rabbit IgG (1:2000, 7074S, Cell Signaling Technology, Inc., Danvers, USA, AB_10697506). All antibodies were diluted in TBST buffer. The membranes were incubated for 1 h at room temperature with primary antibodies, washed three times for 5 min with TBST and then incubated with the secondary antibodies for 45 min. After another three washing steps the membranes were detected with Lumi-Light^PLUS^ Western Blotting Substrate (Roche Diagnostics Deutschland GmbH, Mannheim, Germany) according to manufacturer’s recommendations.

### Live cell imaging

HEK293 cells were imaged 2 days after transfection with fluorescence-tagged HO variant or in co-transfection with HA-SPP or HA-SPP mut at 37°C on a Nikon Ti-E microscope equipped with an incubation chamber (Okolab) using a 60 x oil immersion objective (NA 1.4, Nikon). After exposure to hypoxia (1% O_2_) for 48 h samples only transfected with fluorescence-tagged HO variants were imaged again. A focussed 488 nm laser was used for GFP excitation. GFP emission was measured between 500–550 nm. CFP and YFP were excited with 457 nm and 514 nm, the corresponding emissions were measured between 464-499 nm and 525–555 nm.

### Statistical analysis

Data values of at least three independent experiments were analyzed by one-way ANOVA followed by unpaired student’s t-test versus wild type HO-1 translocation. P-values < 0.05 (*) were considered significant. Data are presented as means with indicated error bars showing +/- SEM.

## Results

### HO-1 proteolysis is SPP-mediated and not found for HO-2

**Fig 1:**
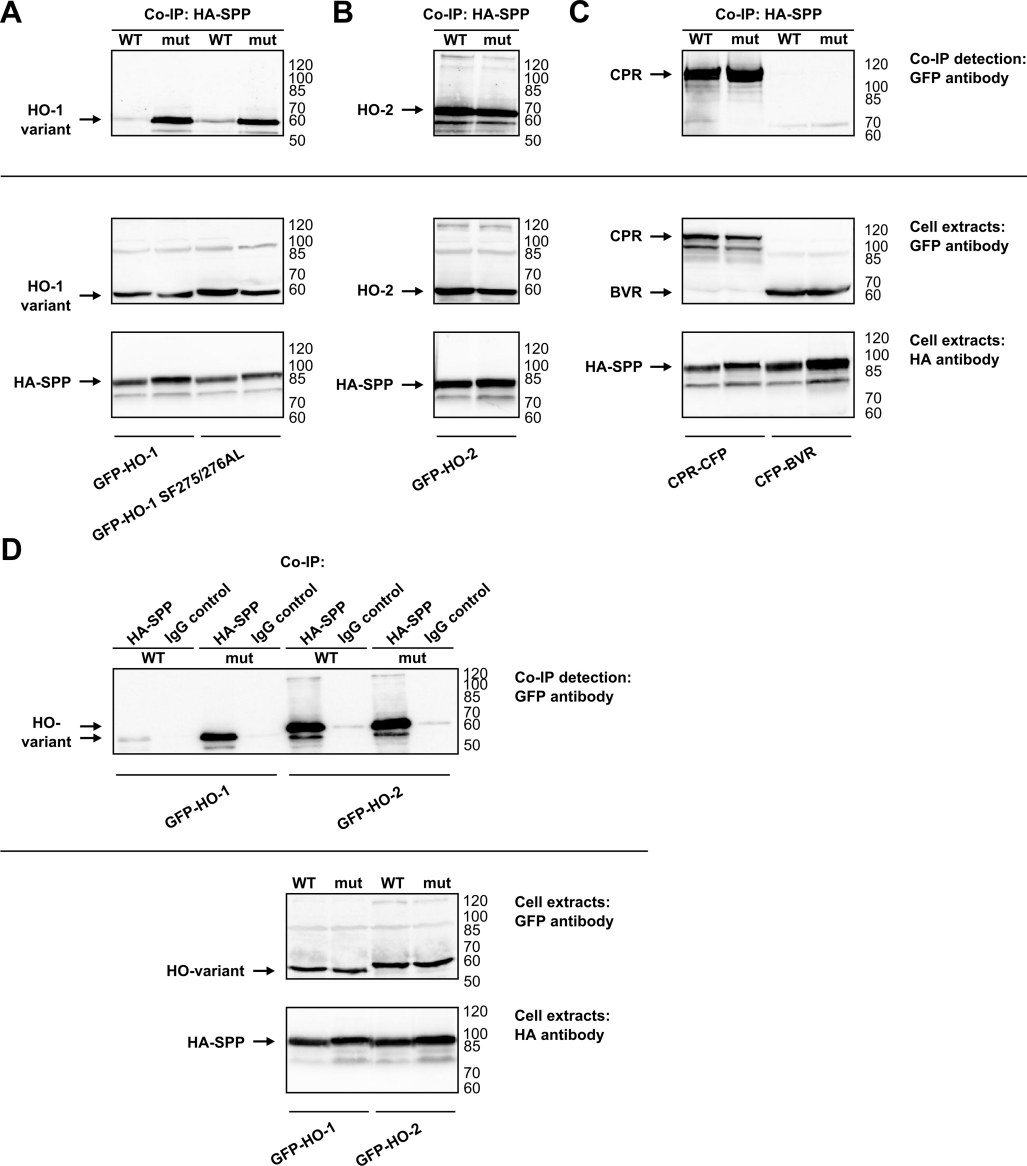
Analysis of SPP binding and cutting of HO variants, CPR and BVR in HEK293 cells by co-immunoprecipitation. Western blots of co-immunoprecipitations (Co-IPs) of fluorescent protein fused HO variants **(A and B)** CPR and BVR **(C)** with HA-antibody, detected with GFP-antibody. **(D)** Western blots of co-immunoprecipitations of fluorescent protein fused HO variants with HA- or IgG control-antibody, detected with GFP-antibody. Western blots of cell extracts used for co-immunoprecipitation were incubated with GFP-antibody to detect HO variants, CPR and BVR and were incubated with HA-antibody to detect SPP as homodimer at around 95 kDa as described by Nyborg et al. [34]. Representative blots of one out of three independent experiments are shown. WT: wild type, mut: inactive mutant, right: ladder [kDa].

To test whether SPP interacts only with HO-1 or also with the homologous HO-2 isoenzyme we performed co-immunoprecipitation experiments (Fig 1A and 1B). HA-tagged SPP and GFP-tagged HO variants were co-expressed in HEK293 cells. Precipitation of the overexpressed SPP with an HA-antibody and subsequent analysis with an GFP-antibody to detect HO variants showed that HO-1 interacts with the catalytically inactive form of SPP (Fig 1A, upper panel). When co-precipitated with catalytically active SPP, the signal for HO-1 was lost. These results were expected as SPP binds to its substrates prior to cleavage and the interaction is lost due to the cleavage reaction [16,17]. Recently, Hsu et al. [17] showed that a HO-1 anchor mutant (SF275/276AL) is partially resistant to SPP-mediated cleavage. In contrast to wild type HO-1 and consistent with the findings of Hsu et al. [17], this mutant was weakly co-immunoprecipitated with active SPP (Fig 1A). The co-precipitation signal for HO-2 was much stronger for both the catalytically active and inactive SPP forms (Fig 1B). This indicates complete SPP cleavage resistance of HO-2 in comparison to only partial SPP cleavage resistance of the SF275/276AL HO-1 mutant. Fig 1C shows co-immunoprecipitations of the catalytically active and inactive SPP with CPR and BVR. CPR, an established interaction partner of both, HO-1 and HO-2, shows a signal with both SPP variants (Fig 1C, upper panel). In contrast, BVR, another HO-interacting protein, neither precipitated with wild type SPP nor with the inactive SPP mutant (see Fig 1C, upper panel). To validate the specificity of our experimental design, corresponding co-immunoprecipitation experiments with HO-1 and HO-2 were performed with an unspecific IgG control-antibody (Fig 1D). While co-immunoprecipitation with HA-antibody showed the same signals as in 1A and 1B, there was no signal for co-immunoprecipitation with the IgG control-antibody. Taken together, these co-immunoprecipitation experiments show that HO-1 is a specific substrate for SPP while HO-2 can interact with SPP without being cleaved.

### HO-1 proteolyis by endogenous SPP occurs only under conditions of hypoxia

**Fig 2:**
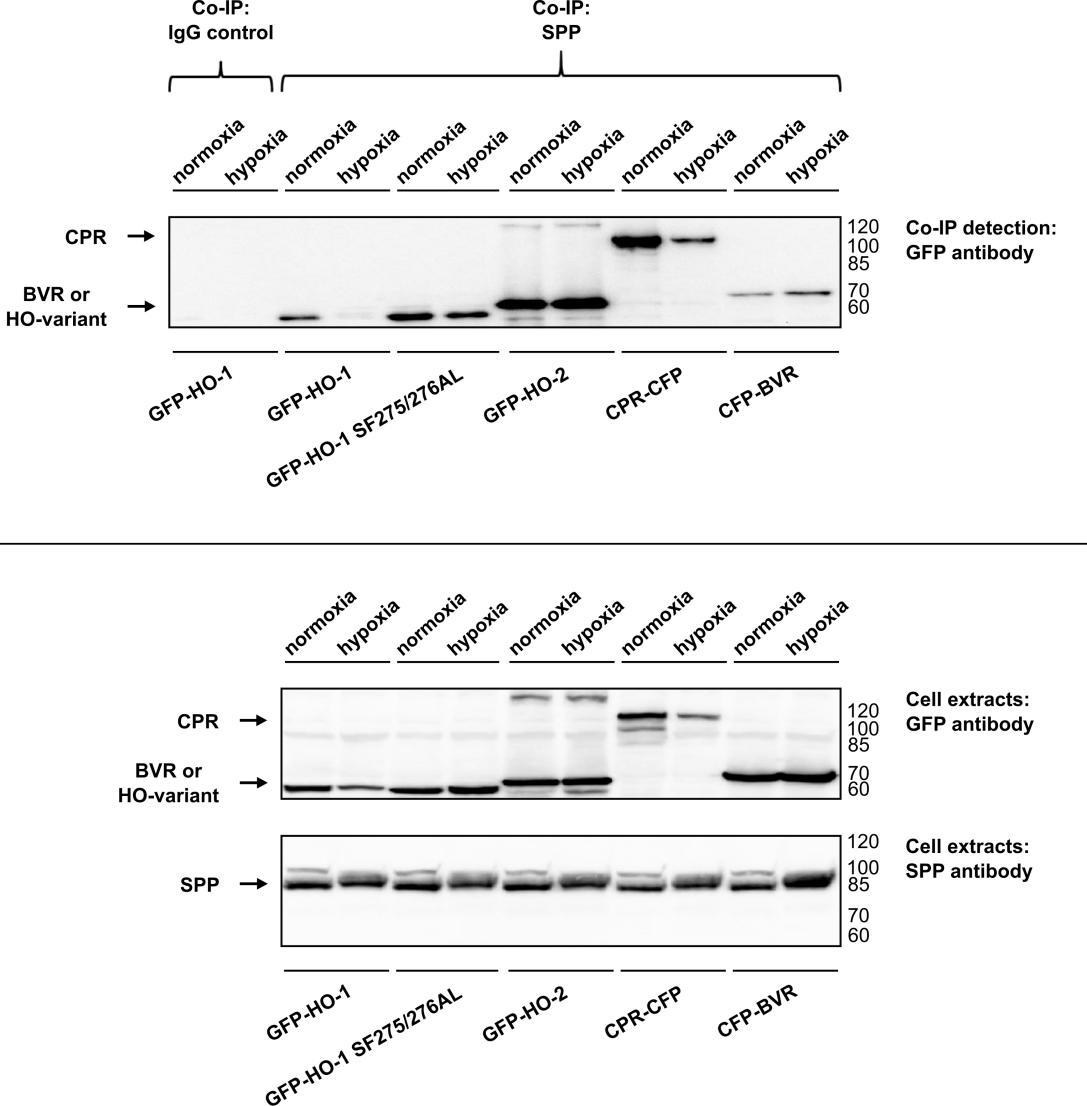
Analysis of endogenous SPP binding and cutting of HO variants, CPR and BVR in HEK293 cells by co-immunoprecipitation under normoxia and after 48 h of hypoxia (1% O_2_). Western blots of co-immunoprecipitations of HO variants, CPR and BVR with SPP-antibody or IgG control-antibody-were detected with GFP-antibody. Western blots of cell extracts used for co-immunoprecipitation were incubated with GFP-antibody to detect HO variants, CPR and BVR and were incubated with SPP-antibody to detect SPP as homodimer. Representative blots of one out of three independent experiments are shown. right: ladder [kDa].

Co-immunoprecipitation experiments of overexpressed HO isoforms were performed using an antibody against endogenous SPP under normoxic and hypoxic conditions. Under normoxic conditions all the fluorescence-tagged HO variants and CPR were detectable with GFP antibody after precipitation with endogenous SPP (Fig 2, upper panel). After 48 h incubation under conditions of hypoxia, the signal for HO-1 disappeared, whereas all other signals stayed the same as under normoxic conditions (Fig 2, upper panel). In contrast to the experiment with overexpressed SPP (Fig 1A), this experiment shows that HO-1 binds to endogenous SPP during normoxia without being cleaved (Fig 2). Cleavage of HO-1 only occurs under conditions of hypoxia. HO-2 and CPR also interact with endogenous SPP under all conditions, while BVR does not interact with SPP (Fig 2, upper panel). There was no signal detectable for co-immunoprecipitation of HO-1 with IgG control-antibody (Fig 2).

### Cell imaging confirms isoform-specific translocation of HO-1 under conditions of hypoxia or co-transfection with SPP

**Fig 3:**
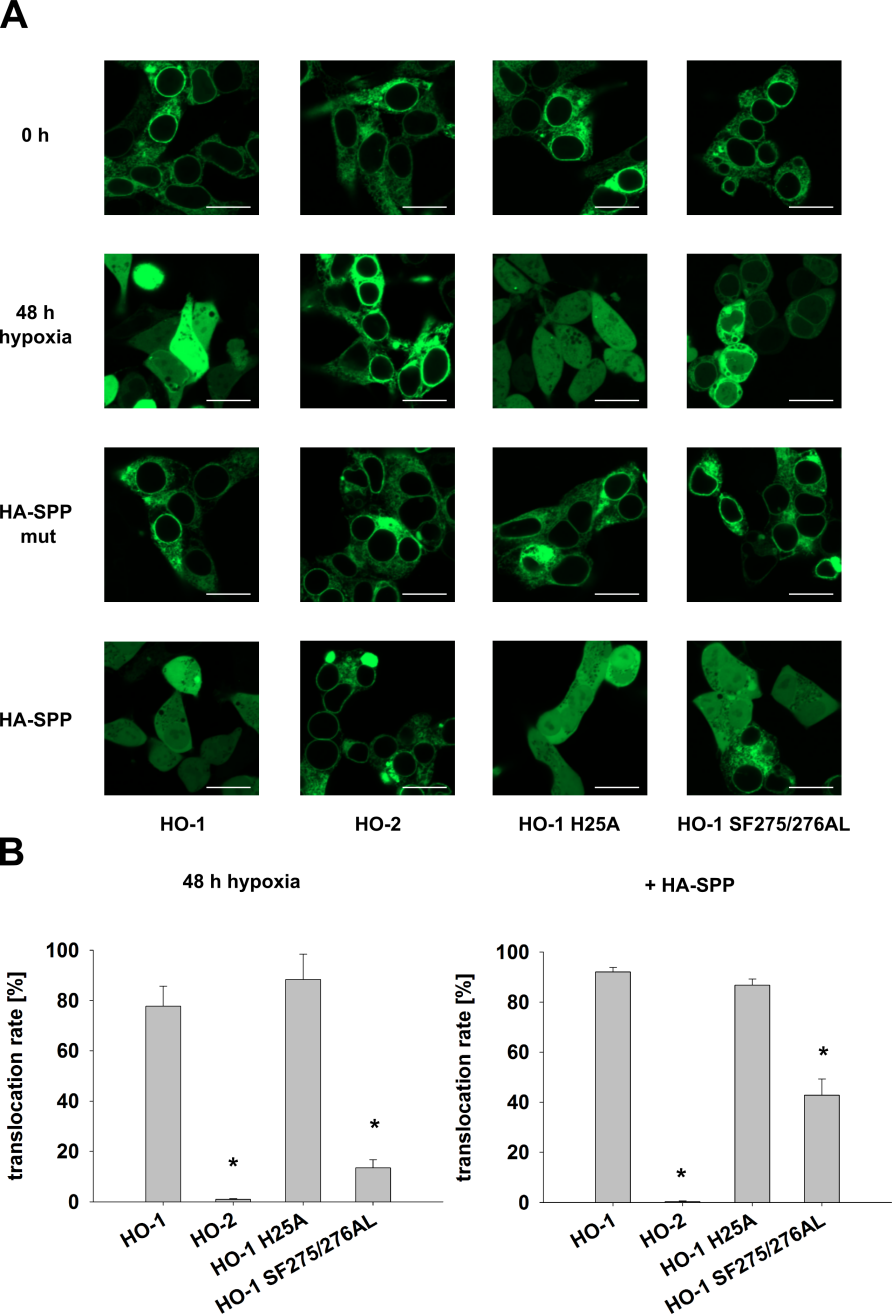
Confocal laser scanning analysis of GFP-tagged HO mutants. HO variants were imaged under normoxic or hypoxic conditions (1% O_2_, 48 h) and co-transfected with wild type HA-tagged SPP or the inactive mutant in HEK293 cells. **(A)** Representative CLSM pictures. Bar represents 20 µm. **(B)** Statistical analysis. Data from five pictures per sample out of three independent experiments was counted and statistically analyzed. Bars: means. Error bars: SEM. * significantly less translocation compared to HO-1 (p < 0.05).

Fig 3A shows that incubation of transfected HEK293 cells under conditions of hypoxia for 48 h leads to translocation of HO-1 but not HO-2. Similarly, co-transfection of SPP leads to translocation of HO-1, but not HO-2. In analogy to co-precipitation experiments catalytically inactive SPP was used as a control. To test whether production of CO or degradation of heme has an influence on the translocation rate, a catalytically inactive HO-1 mutant H25A [30] was tested. In addition the HO-1 anchor mutant SF275/276AL [17] discussed above was tested. In the quantitative analysis shown in Fig 3B five images per sample were taken and the percentages of translocated cells were counted. Catalytic inactivation of HO-1 had no significant influence on the translocation rate neither under conditions of hypoxia nor with SPP co-transfection. In contrast, the anchor mutant showed a significant reduction of translocation under both conditions with an especially pronounced effect under conditions of hypoxia (Fig 3B, for translocation rates in numbers see Table 1). This indicates that the amino acid composition of the ER anchor of HO-1 is of central importance for the isoform-specific HO-1 translocation.

**Table 1:** Translocation rates of HO mutants and chimeras before and after incubation with hypoxia or in co-transfection with HA-SPP.

	48 h hypoxia		+ HA-SPP	
	Translocation (%)	SEM (%)	Translocation (%)	SEM (%)
**HO-1**	**77.72**	**7.90**	**92.06**	**1.74**
**HO-2**	**0.97**	**0.30**	**0.38**	**0.24**
**HO-1 H25A**	**88.26**	**10.14**	**86.72**	**2.52**
**HO-1 SF275/276 AL**	**13.49**	**3.25**	**42.83**	**6.47**
**HO-1-HO-2-anchor**	**0.51**	**0.07**	**2.69**	**0.08**
**HO-2-HO-1-anchor**	**13.01**	**4.85**	**60.93**	**7.68**
**HO-2-HO-1-PEST-anchor**	**38.48**	**5.12**	**95.67**	**0.56**
**HO-2-HO-1-NSS-PEST-anchor**	**72.49**	**5.18**	**89.73**	**0.07**

The rates of cells showing a translocation to the nucleus after incubation with hypoxia (48 h) or in co-transfection with HA-SPP were determined in comparison to the total number of transfected cells. The results consider at least three independent experiments including 5 pictures per experiment and are shown as means (%) ± SEM (%).

### A luminal extension prevents SPP-mediated cleavage of HO-1

**Fig 4:**
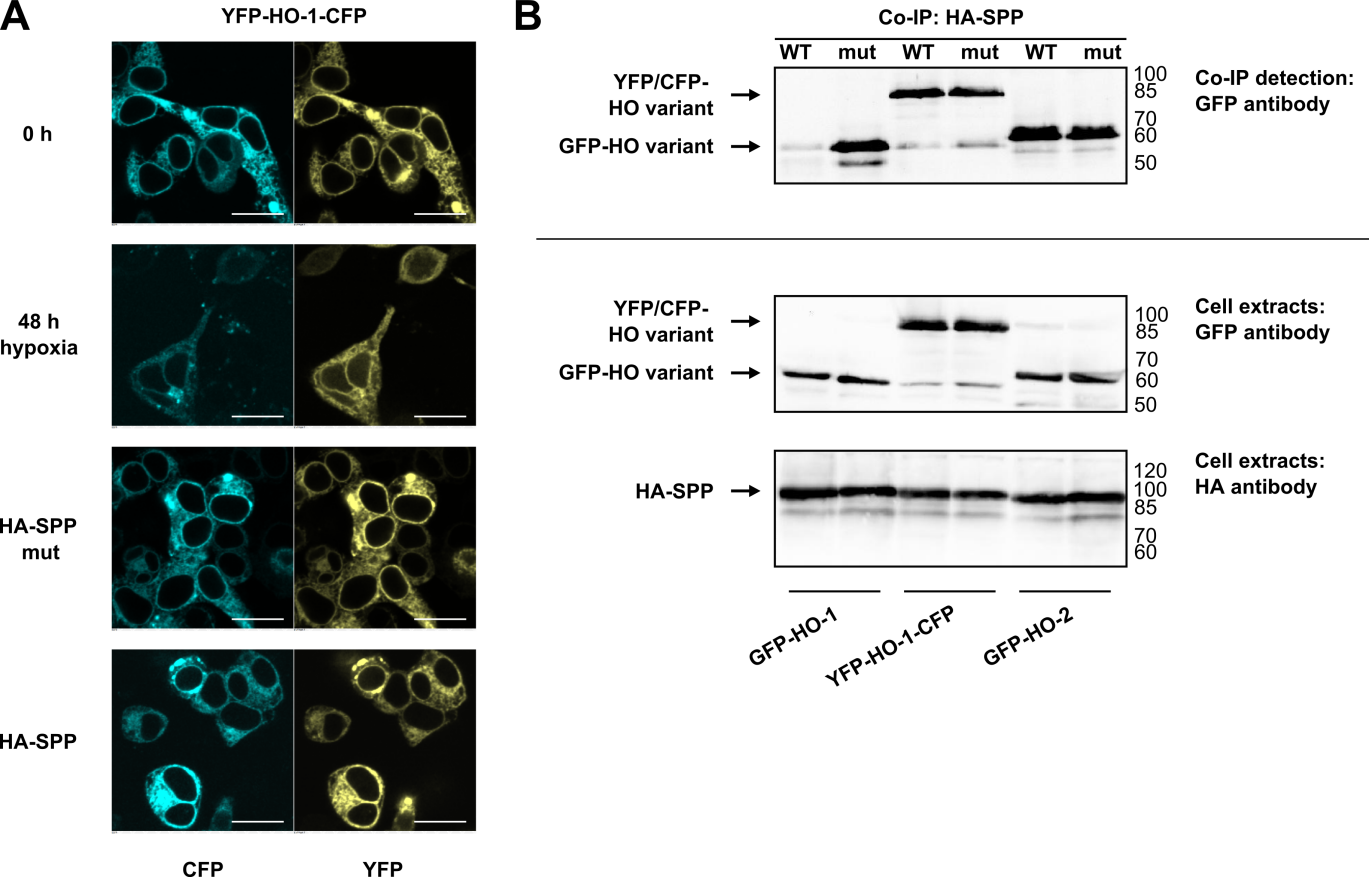
Analysis of SPP interaction of a double fluorescence-tagged HO-1 in HEK293 cells. **(A)** Confocal laser scanning analysis of HO variants under normoxic or hypoxic conditions (1% O_2_, 48 h) and in co-transfection with wild type HA-tagged SPP or the inactive mutant. Figure shows representative data from five pictures per sample out of three independent experiments. Bar represents 20 μm. **(B)** Co-immunoprecipitations with HA-antibody, detected with GFP-antibody. Western blots of cell extracts used for co-immunoprecipitation were incubated with GFP-antibody to detect HO variants and were incubated with HA-antibody to detect SPP as homodimer at around 95 kDa. Representative blots of one out of three independent experiments are shown. WT: wild type, mut: inactive mutant, right: ladder [kDa].

Boname et al. [16] have shown that a luminal GFP-tag prevents SPP-mediated degradation of HO-1. In the current study, we investigated whether limited proteolysis under conditions of hypoxia is also prevented by such a luminal extension within the ER. To test this, we cloned a double fluorescence-tagged HO-1 variant. As shown in Fig 4A by confocal laser scanning microscopy, this HO-1 variant showed no sign of translocation neither under conditions of hypoxia nor in co-transfection experiments of catalytically active SPP. Co-immunoprecipitation showed that the luminal extension prevents limited proteolysis by overexpressed SPP, but does not prevent interaction with SPP (Fig 4B). Our data confirm the results of Boname et al. [16] and suggests that a large luminal extension prevents SPP-mediated cleavage of HO-1, but does not affect SPP binding.

### The hydrophobic carboxy-terminus of HO-1 is necessary, but not sufficient for SPP-mediated translocation

**Fig 5:**
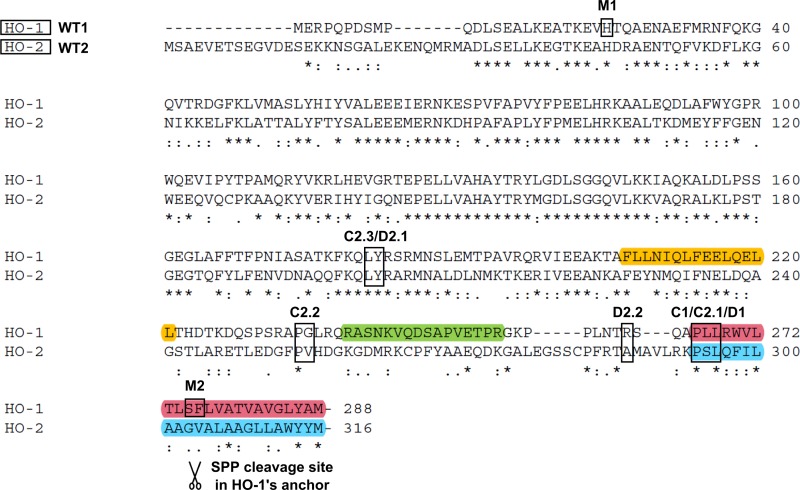
Alignment of human wild type HO-1 and HO-2. Human wild type HO-1 and HO-2 were aligned using Clustal W 2.0 [35]. Wildtype HOs, regions where chimeras shift between HO-1 and HO-2, mutations and beginning or ending of deletion mutants are highlighted. A leucine-rich region or nuclear shuttle sequence (NSS) is shown in yellow, a degradation signal sequence (PEST-domain) in green and the anchor, a lipophilic membrane domain, for HO-1 in red and for HO-2 in blue. HO-1’s anchor contains the SPP cleavage site SF275/276, marked as described in Hsu et al. [17]. Identical amino acids are marked with *, strong and weak conservation is marked with: and. . WT1: Wild type HO-1. WT2: Wild type HO-2. M1: HO-1 H25A. C2.3: HO-2-HO-1-NSS-PEST-anchor. D2.1: HO-1-ΔN181. C2.2: HO-2-HO-1-PEST-anchor. D2.2: HO-1-ΔN262. C1: HO-1-HO-2-anchor. C2.1: HO-2-HO-1-anchor. D1: HO-1-ΔC266. M2: HO-1 SF275/276AL.

**Fig 6:**
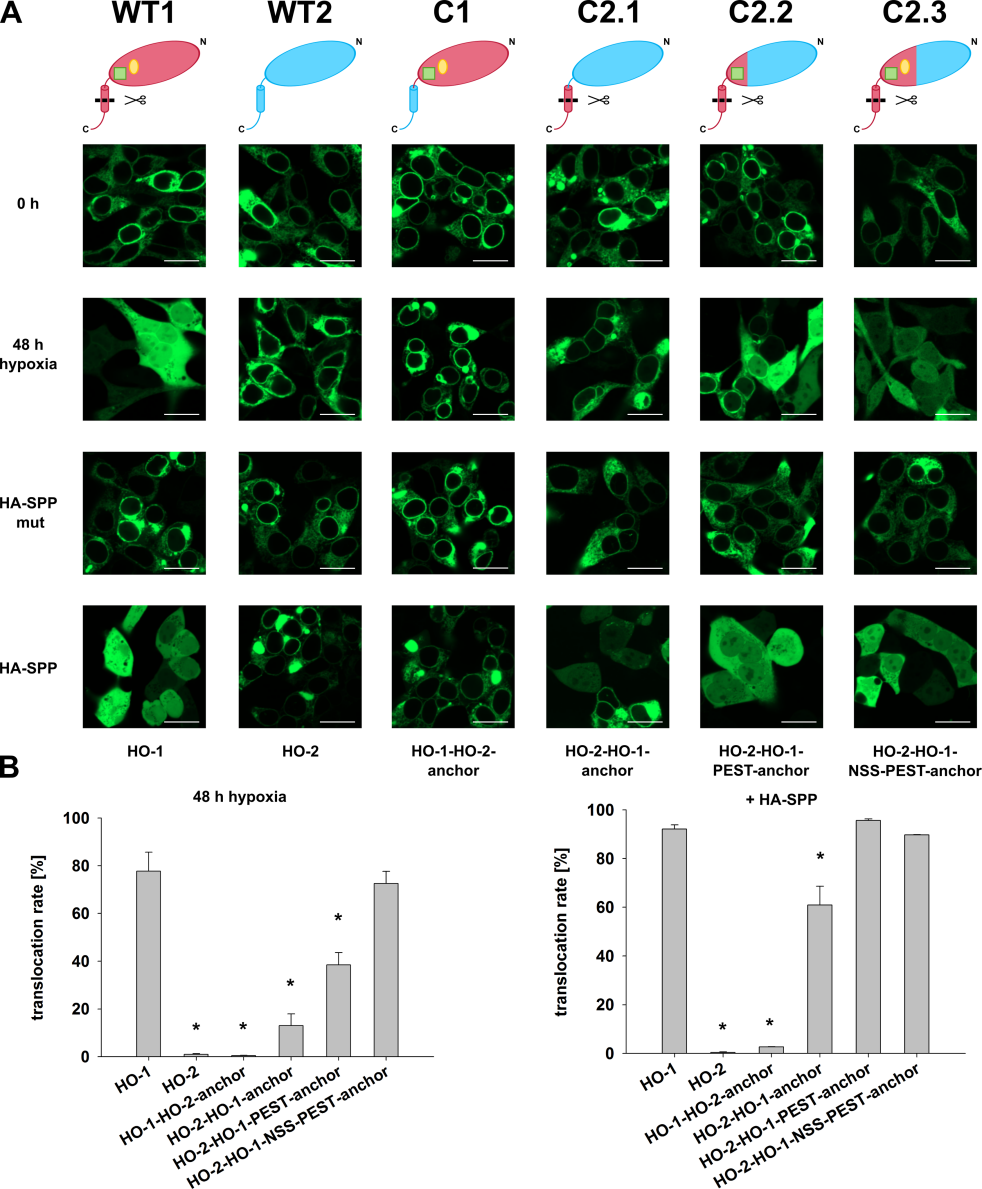
Confocal laser scanning analysis of GFP-tagged HO chimeras. HO variants were imaged under normoxic or hypoxic conditions (1% O_2_, 48 h) and co-transfected with wild type HA-tagged SPP or the inactive mutant in HEK293 cells. **(A)** Representative CLSM pictures. Bar represents 20 μm. WT1: Wild type HO-1. WT2: Wild type HO-2. C1: HO-1-HO-2 chimera. C2.1-C2.3: HO-2-HO-1 chimeras. Models: Red: HO-1 part. Blue: HO-2 part. Green square: PEST domain. Yellow ellipse: NSS. **(B)** Statistical analysis. Data from five pictures per sample out of three independent experiments was counted and statistically analyzed. Bars: means. Error bars: SEM. * significant less translocation compared to HO-1 (p < 0.05).

To further investigate why HO-1 translocates to the nucleus and the cytosol, whereas HO-2 does not, GFP-fused HO-1 / HO-2 chimeras were cloned, transfected into HEK293 cells and imaged by confocal laser scanning microscopy (see detailed information about the HO variants in Fig 5 and cartoons in Fig 6). The translocation behavior of the GFP-fused HO-1 / HO-2 chimeras was compared with GFP-fused wild type HO-1 and wild type HO-2 (Fig 6 WT1 and WT2). Cells were analyzed before and after 48 h incubation under hypoxia or analyzed after co-transfection of catalytically active or inactive HA-tagged SPP (Fig 6, translocation rates in numbers see Table 1).

As expected, wild type HO-1 localizes to the ER membrane and translocates to the cytosol and nucleus after 48 h of hypoxia (Fig 6 WT1). Co-transfection of catalytically inactive SPP led to no change in the ER-localization of HO-1. Co-transfection of catalytically active SPP led to nearly complete translocation of HO-1. In contrast, HO-2 localizes to the ER membrane under all conditions and is completely resistant to cleavage and subsequent translocation under hypoxia or in co-transfection with catalytically active SPP (Fig 6 WT2). HO-1 with an HO-2 ER anchor behaves exactly like HO-2 and neither translocates under conditions of hypoxia nor in co-transfection with SPP (Fig 6 C1). This indicates that the HO-1 ER anchor is necessary for translocation and cleavage by SPP.

The complementary chimera C2.1 consisting of HO-2 with an HO-1 ER anchor led to very low translocation rates under conditions of hypoxia and showed partial translocation in co-expression experiments with catalytically active SPP (Fig 6 C2.1). Increasing the carboxy-terminal part of HO-1 in chimera C2.2 now including HO-1’s PEST domain, which is a protein degradation signal sequence, showed partial translocation under conditions of hypoxia and full translocation in co-transfection experiments with catalytically active SPP (Fig 6 C2.2). Further increase of the carboxy-terminal part of HO-1 now also including the nuclear shuttle sequence (NSS), previously described by Lin et al. [22], led to results identical with wild type HO-1: full translocation under conditions of hypoxia or co-transfection of catalytically active SPP (Fig 6 C2.3).

### The ER anchor of HO-1 is necessary for SPP-binding

**Fig 7:**
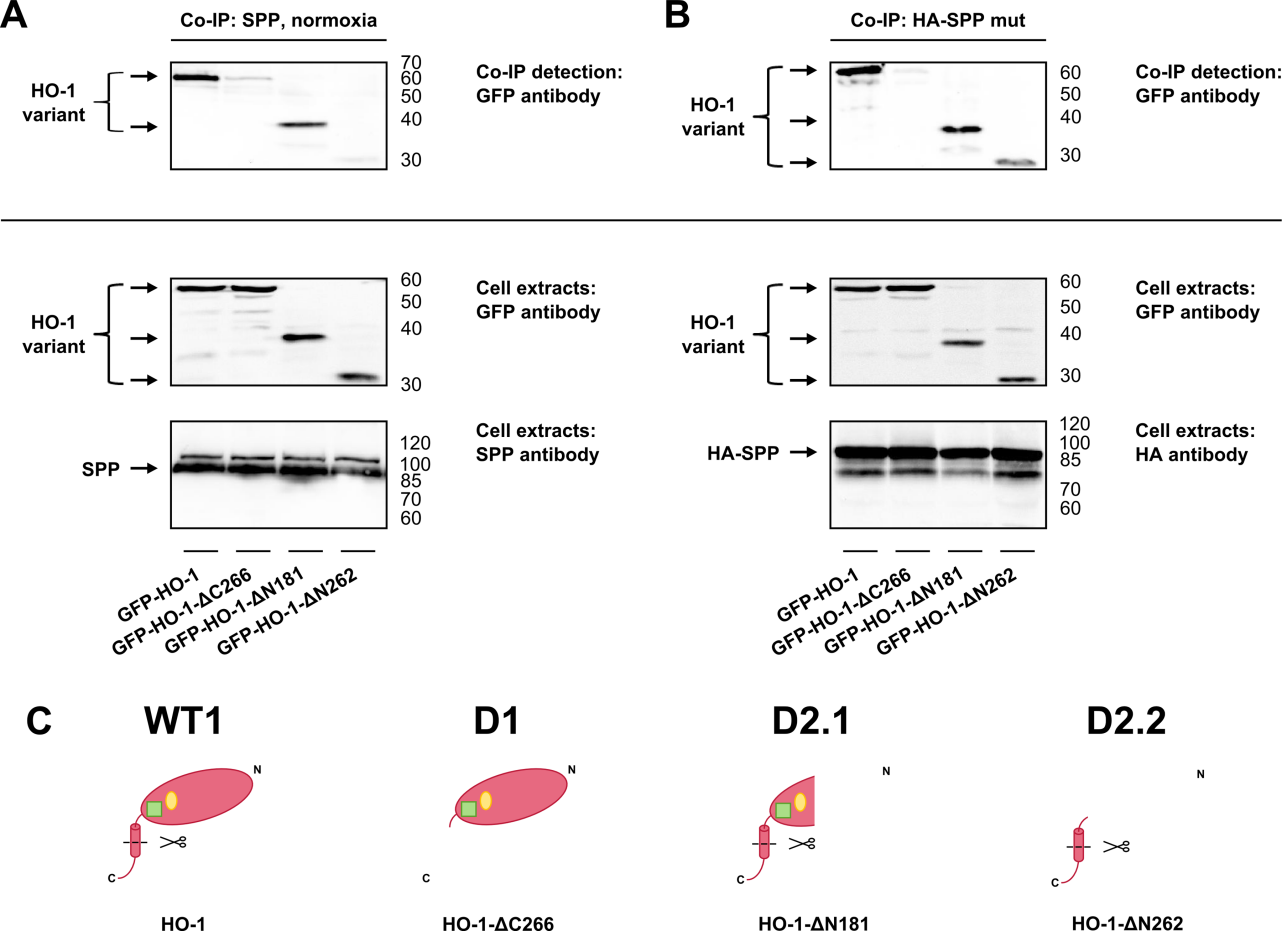
Analysis of SPP binding of HO-1 deletion variants by co-immunoprecipitation. **(A)** HO-1 deletion variants were co-immunoprecipitated with endogenous SPP and detected with anti-GFP-antibody under normoxia. **(B)** HO-1 deletion variants in co-transfection with SPP mut were co-immunoprecipitated with HA-antibody and detected with anti-GFP-antibody. Western blots of cell extracts used for co-immunoprecipitation were incubated with GFP-antibody to detect HO-1 variants and incubated with HA-antibody or SPP-antibody to detect SPP as homodimer. Representative blots of one out of three independent experiments are shown. right: ladder [kDa]. **(C)** Models of the HO-1 deletion variants: WT1: Wild type HO-1. D1: HO-1-ΔC266. D2.1: HO-1-ΔN181. D2.2: HO-1-ΔN262. Red: HO-1 part. Green square: PEST domain. Yellow ellipse: NSS.

To validate the results of the chimera studies and to determine the sequences of HO-1 necessary for SPP-binding, we performed co-immunoprecipitation experiments with HO-1 deletion variants (Fig 7C, see Fig 5 for a more detailed description of the variants). While the anchorless HO-1 variant and the isolated anchor could not be precipitated with endogenous SPP, full length HO-1 and a HO-1 variant containing PEST domain and NSS could interact with SPP (Fig 7A). In the presence of overexpressed inactive SPP the HO-1 anchor, but not the anchorless variant is sufficient for co-immunoprecipitation with SPP (Fig 7B). We conclude, that the ER anchor of HO-1 is crucial for SPP binding.

### SPP’s PAL motif is not responsible for substrate binding

**Fig 8:**
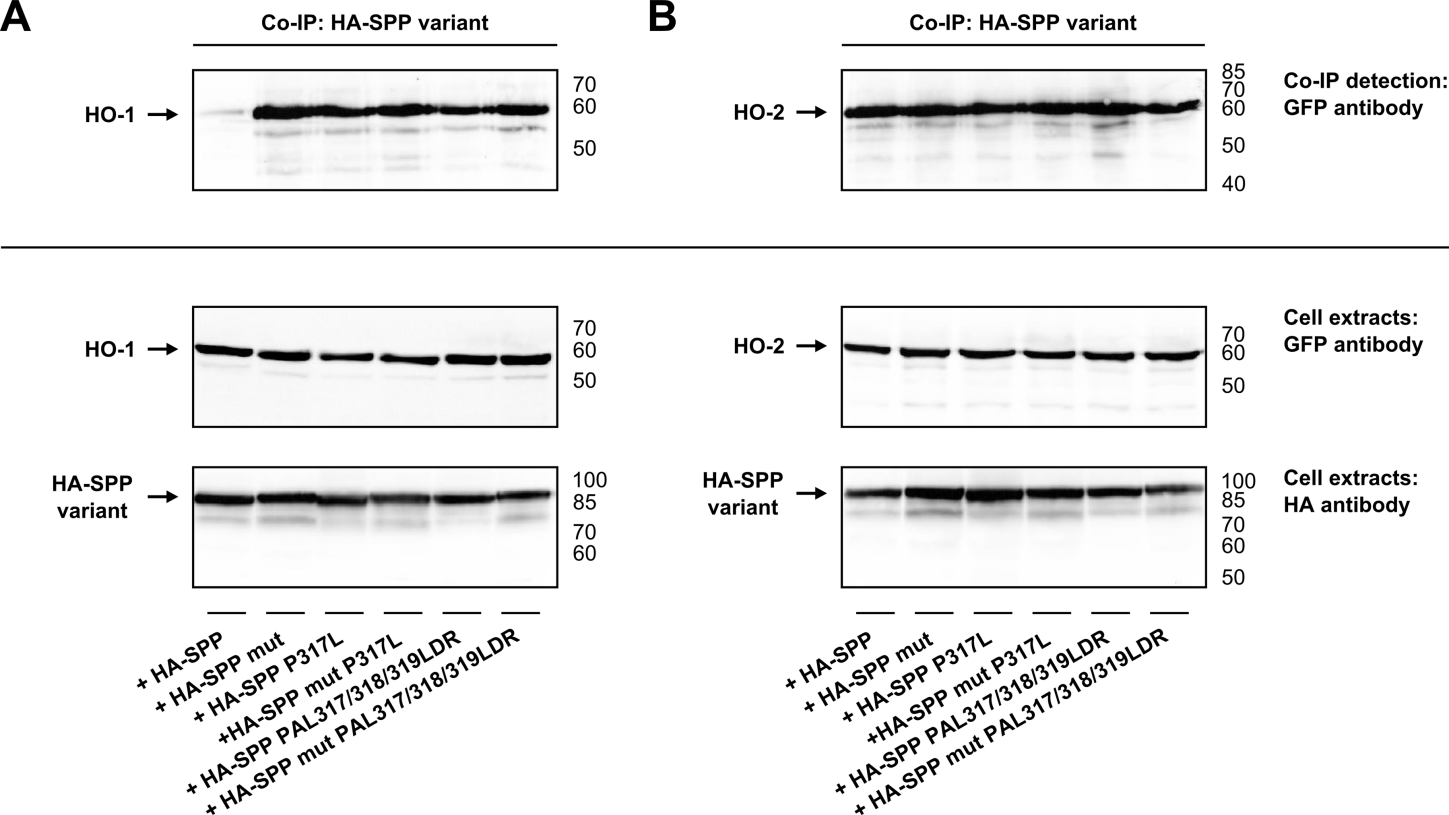
Binding analysis of SPP PAL mutants to wild type HO-1 and wild type HO-2 by co-immunoprecipitation. **(A)** HO-1. **(B)** HO-2. Both show Western blots of co-immunoprecipitations of PAL mutants of SPP and HO-1 or HO-2 with HA-antibody, detected with GFP-antibody. Western blots of cell extracts used for co-immunoprecipitation are incubated with GFP-antibody to detect HO-1 or HO-2 and HA-antibody to detect SPP as homodimer. Representative blots of one out of three independent experiments are shown. right: ladder [kDa].

As HO-1’s sequences involved in SPP binding and cleavage could be identified, we investigated the PAL motif of SPP, a potential substrate binding site [36]. For the function of the PAL motif it is important to know its localization in the secondary structure of SPP, but until now there exists no crystal structure of SPP. That is why we used secondary structure prediction tools for localizing the PAL motif within the SPP. The PSIPRED server [37] predicts the PAL motif within a loop in front of the last transmembrane domain (TMD) of SPP, while the TMHMM server [38] predicts the PAL motif in the last TMD of SPP (see complete predictions in the supporting information). To experimentally investigate the role of the PAL motif, HO isoenzymes were co-immunoprecipitated with PAL mutants of HA-SPP and HA-SPP mut by HA-antibody (Fig 8A and 8B). GFP-antibody was used for detection. As described before [16,17] there is no signal for HO-1 in Fig 8A, because wild type HA-SPP cleaves HO-1. In Fig 8B catalytically active SPP binds to HO-2 without cleaving it, as already described in Fig 1B. HA-SPP mut, which contains a mutation in its active site, binds to both isoenzymes, as shown before (Fig 1A and 1B). All SPP variants with mutations in the PAL motif interact with HO-1 and HO-2, but substrate cleavage could not be observed. Hence a mutation in the PAL sequence leads to catalytically inactive SPP, as already described [39], but does not interfere with substrate binding.

## Discussion

SPP shows no clear consensus cleavage site based on the primary amino acid sequence of its substrates. Nevertheless, a common denominator among SPP substrates is a helix destabilizing residue in the transmembrane helix and a type II-like orientation within the ER with the carboxy-terminus on the luminal side [40,36]. We have previously shown that heme oxygenase isoforms HO-1 and HO-2 differ in their subcellular trafficking during hypoxia [23]. In the current study, we show that HO-1 is cut by SPP while HO-2 is highly resistant to SPP-cleavage. Analysis of the site of SPP-cleavage in the HO-1 membrane anchor and comparison with the respective sequence in HO-2 shows that the hydrophobic transmembrane helix in HO-1 is interrupted by a polar serine residue (S275) at the site of SPP-cleavage [17], while no such polar amino acid can be found in the HO-2 anchor. This is consistent with Boname et al. [16], who suggest that the degradation signal for SPP cleavage of HO-1 is likely imparted in the transmembrane region and Hsu et al. [17], who show that mutation of S275 leads to a reduced translocation. Nevertheless, our finding that an HO-2 chimera with an HO-1 membrane anchor is a poorer SPP substrate than wild type HO-1 points to an influence of additional elements in HO-1. The PEST sequence and NSS present in HO-1 and absent in HO-2 are immediately adjacent to the transmembrane region. These sequences likely influence the strength of the alpha-helical conformation of the HO-1 transmembrane region. Alternatively, the presence or absence of these domains may mediate protein-protein interactions or regulate the extent of oligomerization. Self-interaction may shield the HO transmembrane regions from proteolysis by SPP. Similarly, interaction with another protein could also prevent HO-1 cleavage.

Boname et al. observed that a luminal fluorescence-tagged HO-1 is prevented from cleavage by overexpressed SPP [16]. This is consistent with the literature, as physiological cleavage of signal peptides by SPP requires luminal signal peptidase cleavage in advance to separate the signal peptides from their corresponding proteins [3]. It might have been reasonable to assume that SPP and HO-1 cannot interact, because the luminal tag is too large. However, our results indicate that a large luminal extension still allows SPP to bind to HO-1. Hence there must be other explanations for cleavage protection. Probably the luminal tag stabilizes the anchor of HO-1, so that its helix is not accessible for SPP cleavage [3]. Stabilization of a binding partner by interaction with SPP was already observed by Schrul et al. for the SPP interaction with misfolded opsin [3,32]. Thus a large luminal extension protects from SPP cleavage and subsequent translocation, but still allows SPP to bind.

HO-1 as SPP substrate and HO-2 as SPP binding partner were used to investigate whether the PAL motif of SPP plays a role in substrate binding as proposed by Voss et al. [36]. It is known so far, that mutations in the PAL motif inhibit the enzymatic activity of SPP and that they abolish inhibitor binding [39], but whether PAL mutants are still able to bind to other proteins remained unclear. The localization of a motif in the secondary structure of a protein may help to explain its function. As there is no crystal structure of SPP available, we used two secondary structure prediction tools, the PSIPRED server [37] and the TMHMM server [38], to predict the probable localization of the PAL motif in the three-dimensional structure of SPP. The PSIPRED server [37] predicts the PAL motif in a loop in front of the last TMD of SPP compatible with a role in active site architecture and catalysis. The TMHMM server [38] predicts the PAL motif in the last TMD of SPP compatible with a role in substrate binding. In our experiments PAL mutants of SPP still bound to HO-1 and HO-2, but no cleavage was observed (Fig 8). This indicates that the PAL motif of SPP plays a role in catalysis or active site architecture. Hence, a localization in the loop in front of the last TMD as predicted by the PSIPRED server [37] is more likely than in the last TMD.

Classical SPP substrates such as the signal sequence of preprolactin are processed by SPP as soon as they become available after cleavage from preprolactin by signal peptidase. Translocation of HO-1 to the cytosol and nucleus by SPP has been described as a process that is induced by hypoxia or by cell stress or occurs with progressive malignant progression in cancer or leukemia cells [17,26,28,41]. This indicates that translocation of HO-1 by SPP is a regulated process that could be either induced by changes in HO-1 confirmation or by changes in SPP that result in a general SPP activation or a broadening of SPP substrate specificity to include HO-1.

The first alternative would be a change in HO-1 as the substrate. A crucial co-substrate for the heme oxygenase reaction is oxygen, and HO-2 has even been suggested to act as an oxygen sensor [42,43] although this is somewhat controversial [44]. In the current study, we found that an enzymatically inactive HO-1 variant, created by mutation of histidine 25 into alanine [30,45,46], still translocates to the cytosol and nucleus under conditions of hypoxia. This argues against the idea that translocation under hypoxia is induced by a lack of enzyme activity due to the lack of the co-substrate oxygen. Our finding that overexpressed SPP leads to translocation of HO-1 without a need of hypoxia, also argues against the idea that a major change is induced in the substrate HO-1 by hypoxia.

The second alternative would be a general change in the expression of SPP. Given the diversity of endogenous SPP substrates in a cell, such a general upregulation of enzyme activity also seems rather unlikely. In line with this, there is currently no information in the literature pointing to a hypoxia induced increase in SPP levels and / or SPP activity. In our model system, we did also not find any upregulation of SPP in response to hypoxia (Fig 2 lower panel). However, we did detect a difference in the signal of endogenous SPP between normoxia and hypoxia in Western blots when hypoxia and normoxia samples were run next to each other on a gel. While SPP could be detected as a clear double band of the SPP homodimer under normoxic conditions, as described by Nyborg et al. 2004 [34], the signal looked different after incubation under hypoxia (Fig 2). This change in the SDS gel migration behavior between the SPP homodimer may reflect a posttranslational modification of SPP induced by hypoxia. It is tempting to speculate that this posttranslational modification either activates SPP or broadens the substrate specificity to include HO-1 or HO-1-like proteins in addition to signal sequences. Whether the change in migration behavior really reflects meaningful regulation is an interesting research project for the future.

If this change in running behavior of SPP turns out to be an artifact and if it is not the substrate and not the protease SPP, a third protein or protein complex could also be involved: XBP1, an unfolded protein response regulator, is a substrate for SPP, that requires interaction with Derlin1 and TRC8 for SPP cleavage [47]. In this case Derlin1 seems to bind to the luminal substrate tail thereby obviating the need for ectodomain shedding prior to SPP cleavage. While HO-1 has no luminal substrate tail that inhibits cleavage by SPP and might be regulated by Derlin-1, HO-1 has been reported to interact with TRC8 from the above mentioned complex [48]. The ER-located E3 ligase TRC8 has been shown to target HO-1 for ubiquitination and degradation [48,49], but has not been linked to SPP-mediated HO-1 translocation so far. While it has been shown that SPP-mediated translocation of HO-1 to the cytosol and nucleus promotes cancer cell proliferation and invasion [17,27], targeting HO-1 for ubiquitination and degradation by TRC8 is thought to suppress tumorigenesis [48]. Studying the isoform specificity of TRC-8 mediated HO-ubiquitination represents a complex research project on its own that is very interesting but beyond the scope of the current paper.

We believe that the difference between both HO isoforms together with the HO chimeras characterized in the current paper, will not only be useful for further analysis of SPP substrate binding and catalysis. The chimeras can also be used for studying the HO-TRC8 interaction leading to HO ubiquitination, degradation and tumor suppression [48]. It will be important to determine under what circumstances HO-1 suppresses tumor growth and under what conditions it translocates to the nucleus thereby promoting tumor growth and drug resistance. The current study suggests that avoidance of tumor hypoxia may help to limit HO-1 translocation mediated tumor progression.

## Supporting information

S1 File Uncropped images of Western blots from Fig 1A–1C. **Analysis of SPP binding and cutting of HO variants, CPR and BVR in HEK293 cells by co-immunoprecipitation.** Black boxes show cropped regions.
(TIFF)

S2 File Uncropped images of Western blots from Fig 1D. **Analysis of SPP binding and cutting of HO variants in HEK293 cells by co-immunoprecipitation with HA- and IgG control-antibody.** Black boxes show cropped regions.
(TIFF)

S3 File Uncropped images of Western blots from Fig 2. **Analysis of endogenous SPP binding and cutting of HO variants, CPR and BVR in HEK293 cells by co-immunoprecipitation.** Black boxes show cropped regions.
(TIFF)

S4 File Uncropped images of Western blots from Fig 4B. **Analysis of SPP interaction of a double fluorescence-tagged HO-1 in HEK293 cells.** Black boxes show cropped regions.
(TIFF)

S5 File Uncropped images of Western blots from Fig 7. **Analysis of SPP binding of HO-1 deletion variants by co-immunoprecipitation.** Black boxes show cropped regions.
(TIFF)

S6 File Uncropped images of Western blots from Fig 8. **Binding analysis of SPP PAL mutants to wild type HO-1 and wild type HO-2 by co-immunoprecipitation.** Black boxes show cropped regions.
(TIFF)

S7 File Structural prediction of SPP (PSIPRED server). (TIFF)

S8 File Structural prediction of SPP (TMHMM server). (TIFF)
