# Supplementary figures and images for: Insights into the mechanism of isoenzyme-specific signal peptide peptidase-mediated translocation of heme oxygenase
Source: PLoS One. 2017 Nov 20;12(11):e0188344. doi: 10.1371/journal.pone.0188344 (PMC5695791; doi:10.1371/journal.pone.0188344)

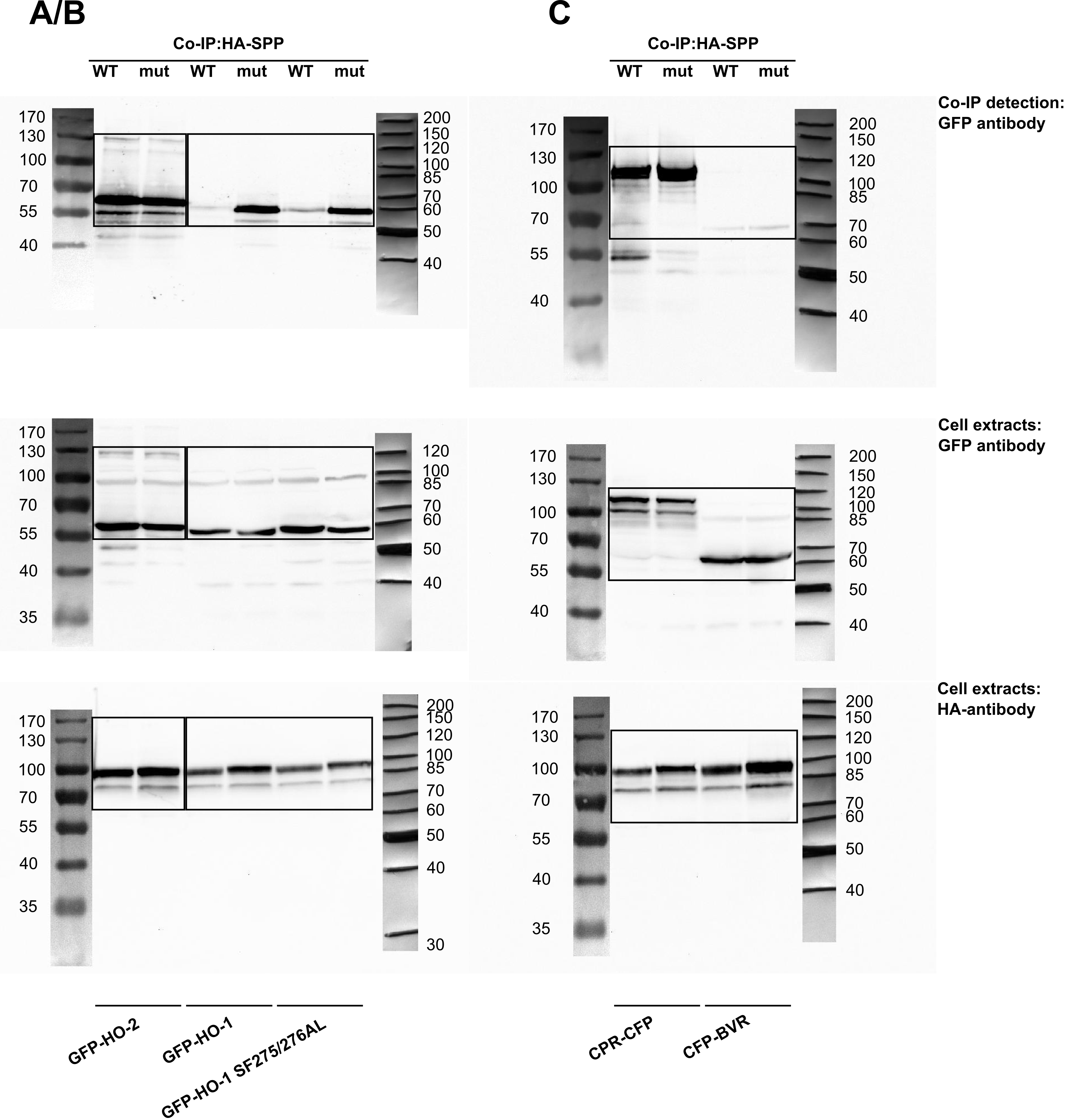

Supplement: S1 File — Analysis of SPP binding and cutting of HO variants, CPR and BVR in HEK293 cells by co-immunoprecipitation. Black boxes show cropped regions. (TIFF) [file pone.0188344.s001.tiff]

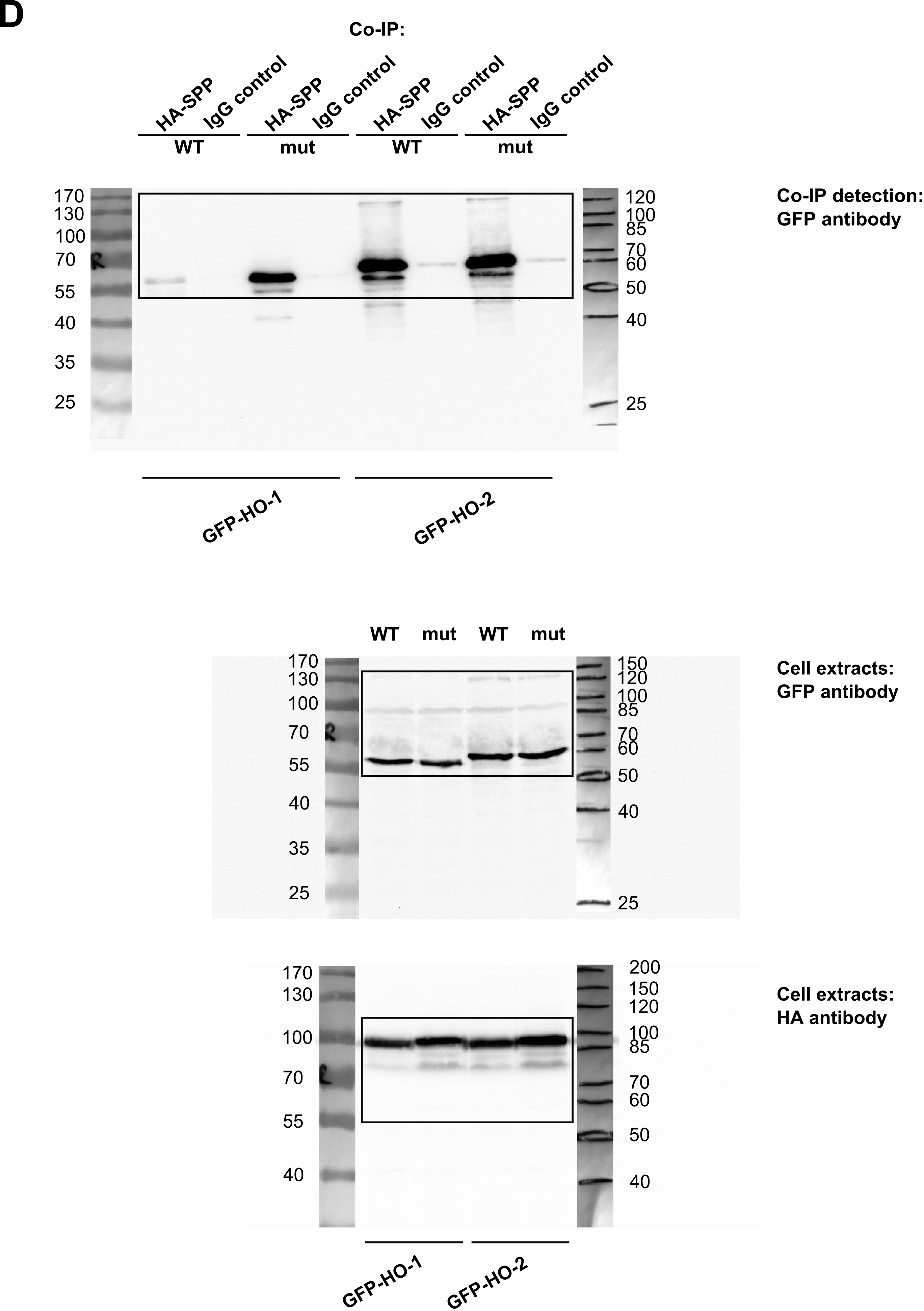

Supplement: S2 File — Analysis of SPP binding and cutting of HO variants in HEK293 cells by co-immunoprecipitation with HA- and IgG control-antibody. Black boxes show cropped regions. (TIFF) [file pone.0188344.s002.tiff]

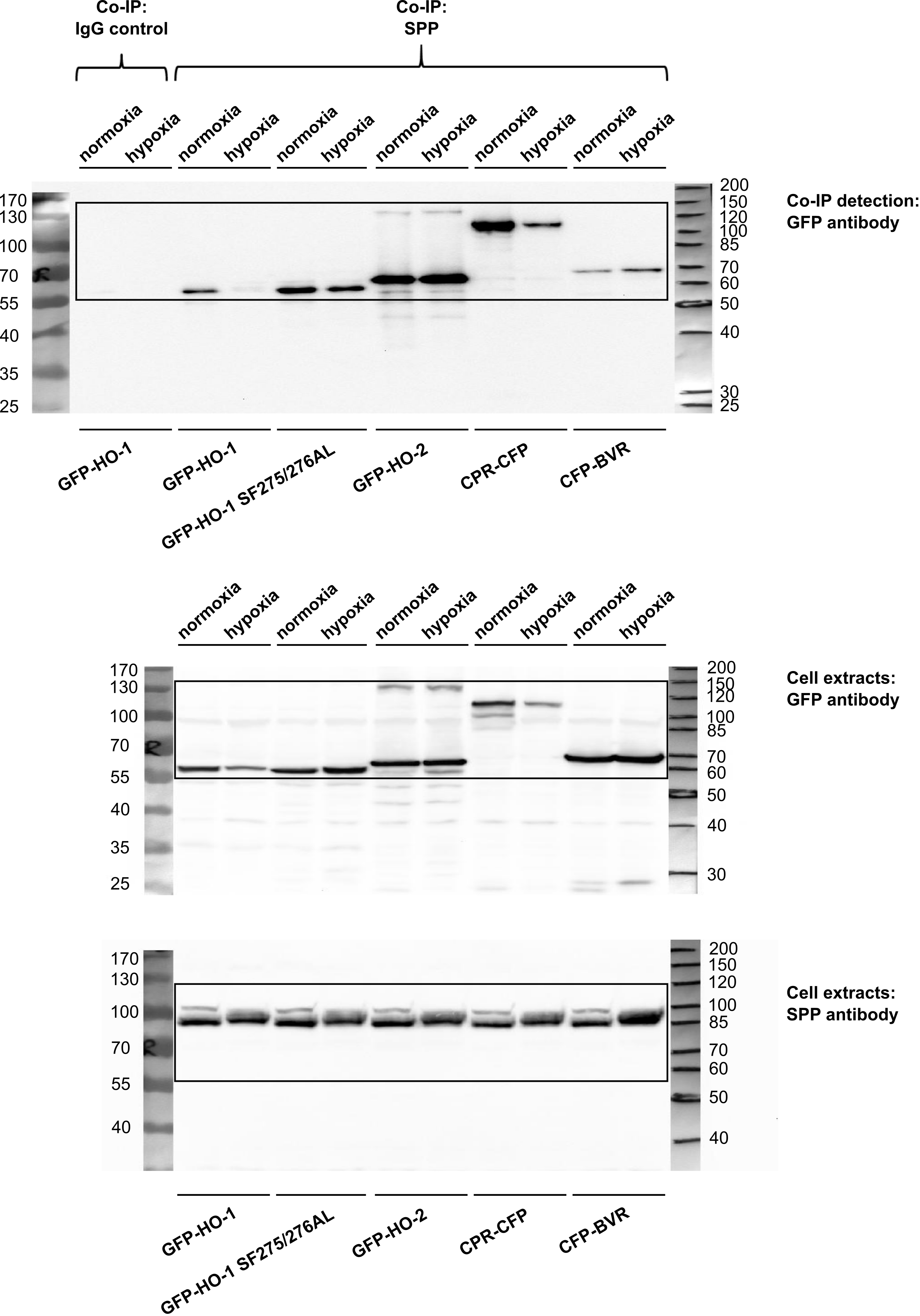

Supplement: S3 File — Analysis of endogenous SPP binding and cutting of HO variants, CPR and BVR in HEK293 cells by co-immunoprecipitation. Black boxes show cropped regions. (TIFF) [file pone.0188344.s003.tiff]

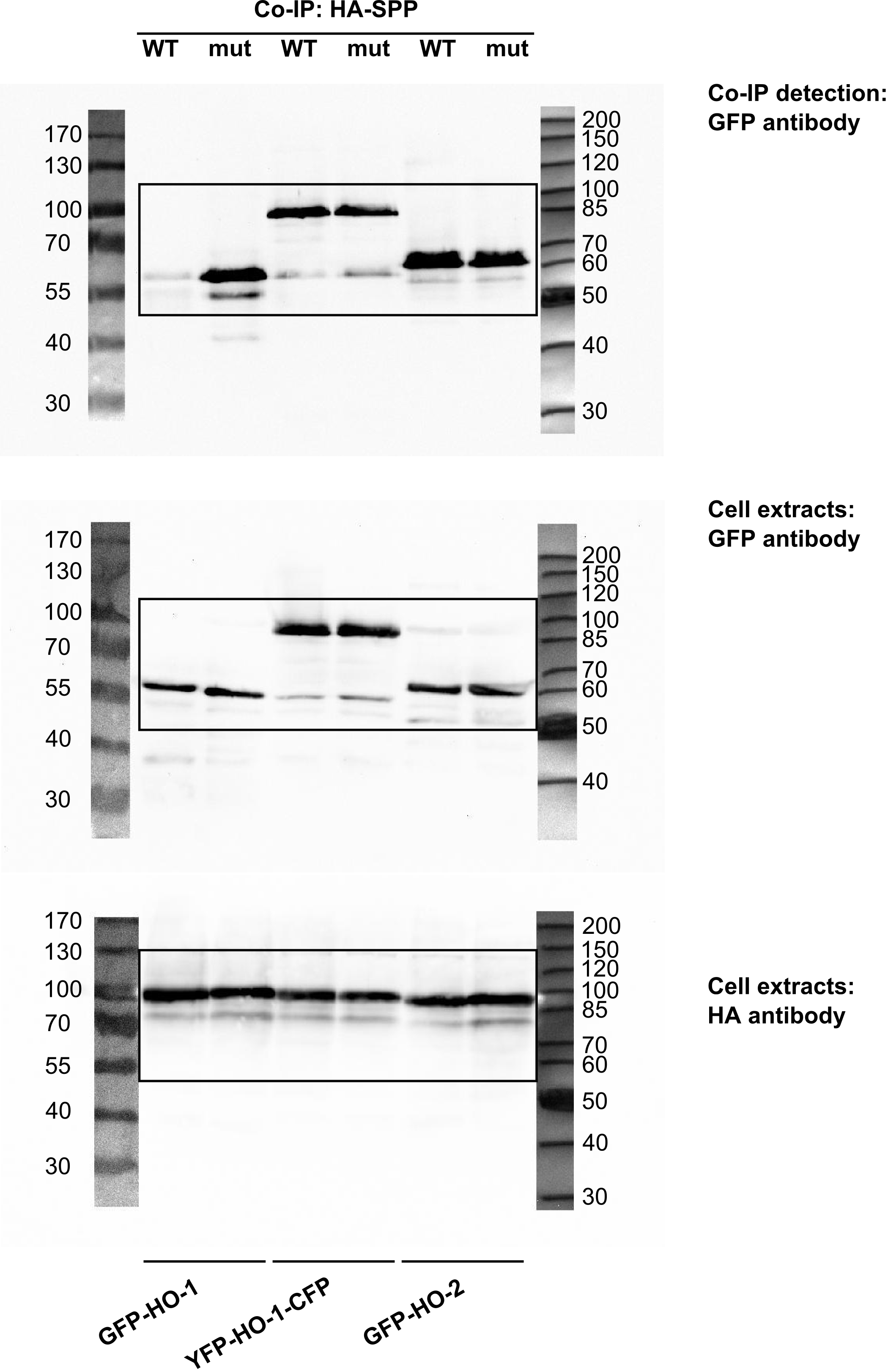

Supplement: S4 File — Analysis of SPP interaction of a double fluorescence-tagged HO-1 in HEK293 cells. Black boxes show cropped regions. (TIFF) [file pone.0188344.s004.tiff]

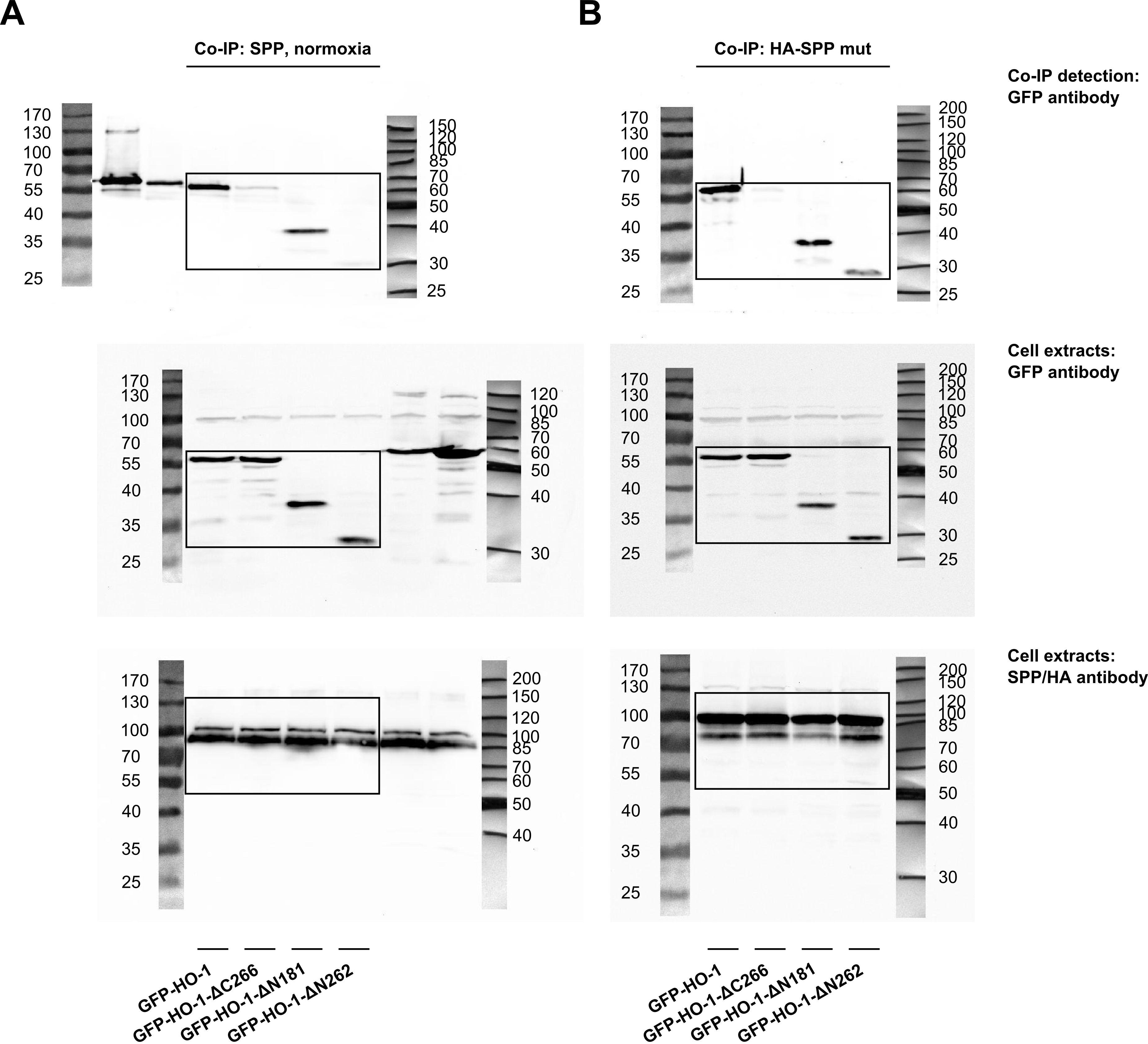

Supplement: S5 File — Analysis of SPP binding of HO-1 deletion variants by co-immunoprecipitation. Black boxes show cropped regions. (TIFF) [file pone.0188344.s005.tiff]

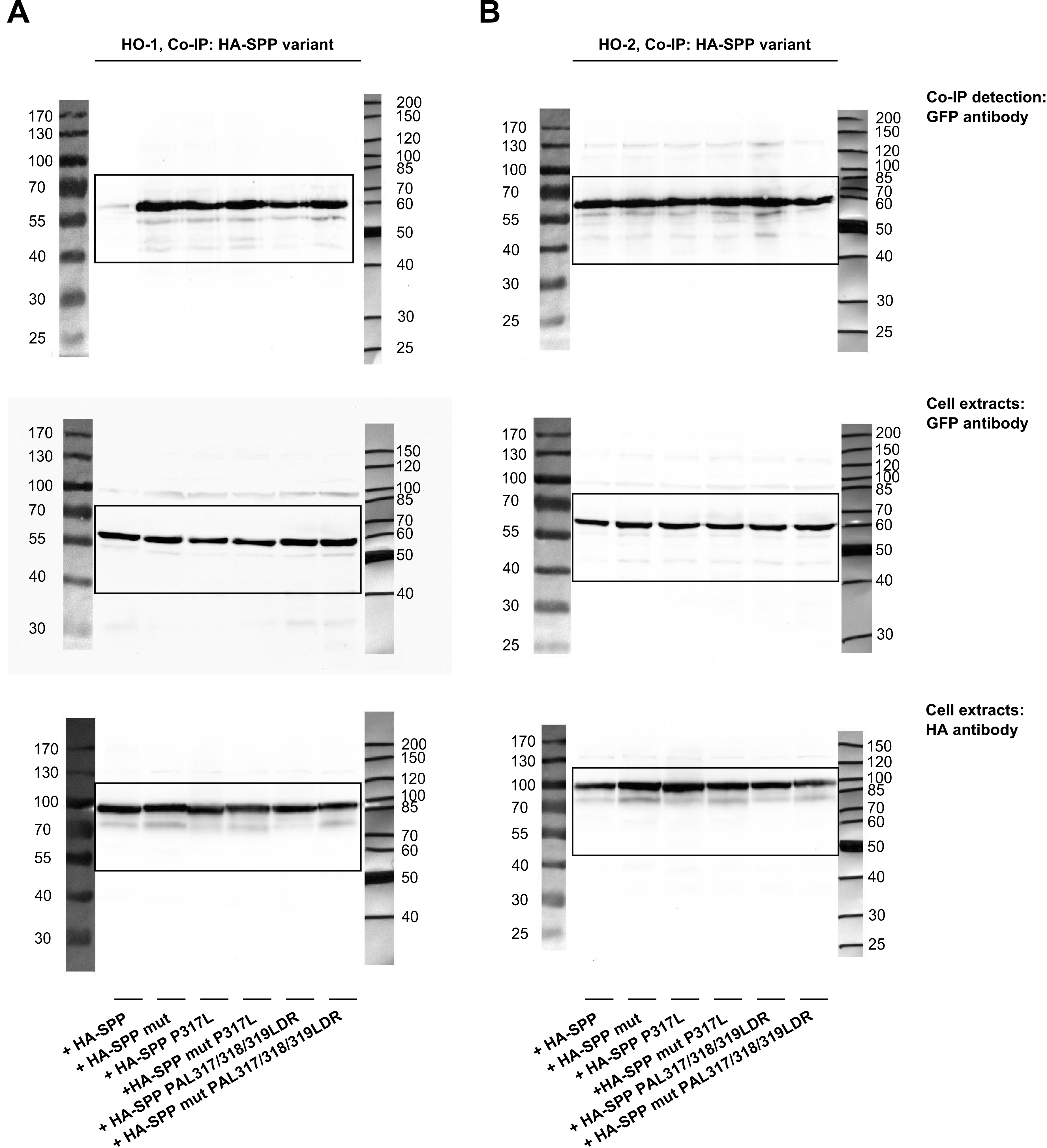

Supplement: S6 File — Binding analysis of SPP PAL mutants to wild type HO-1 and wild type HO-2 by co-immunoprecipitation. Black boxes show cropped regions. (TIFF) [file pone.0188344.s006.tiff]

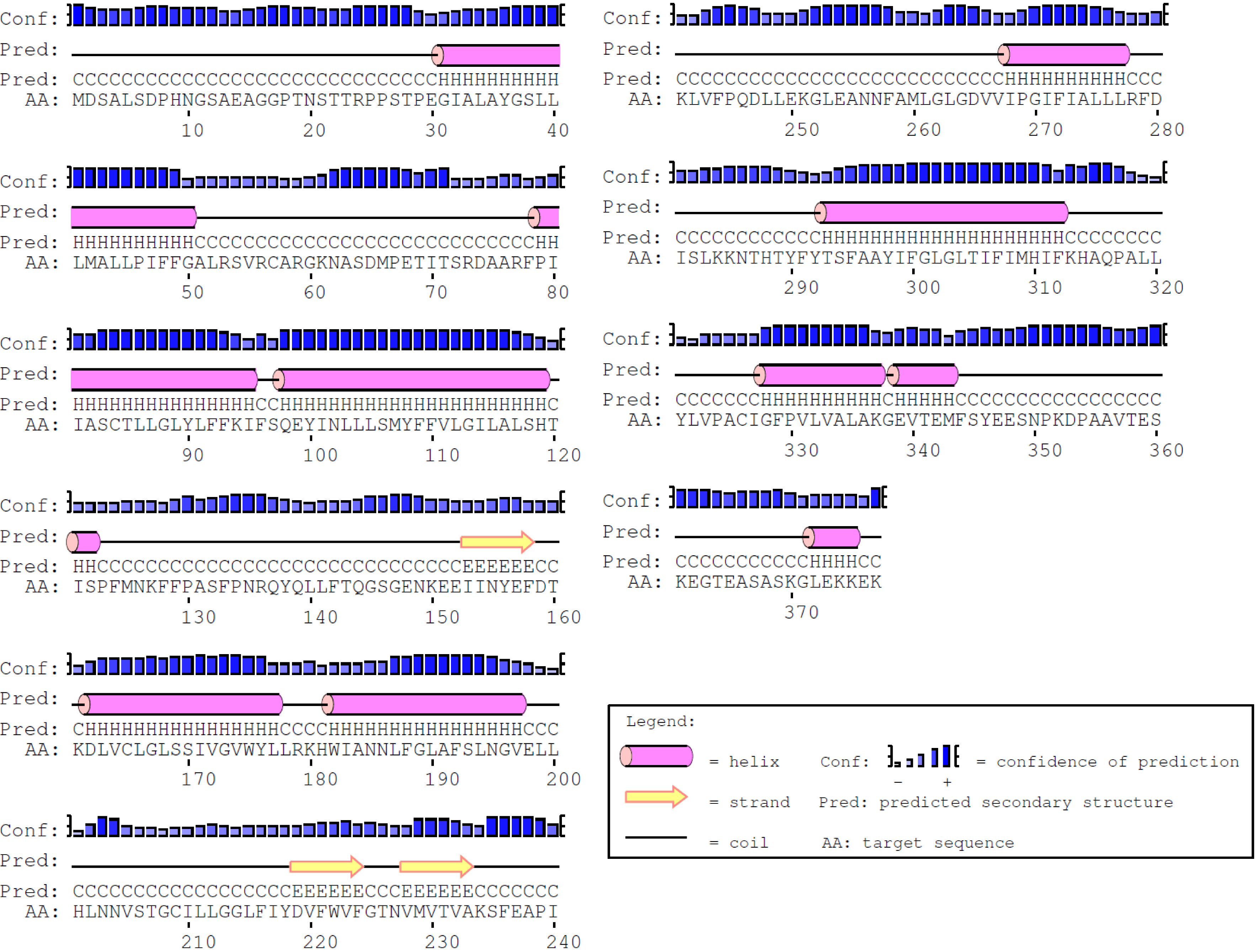

Supplement: S7 File — (TIFF) [file pone.0188344.s007.tiff]

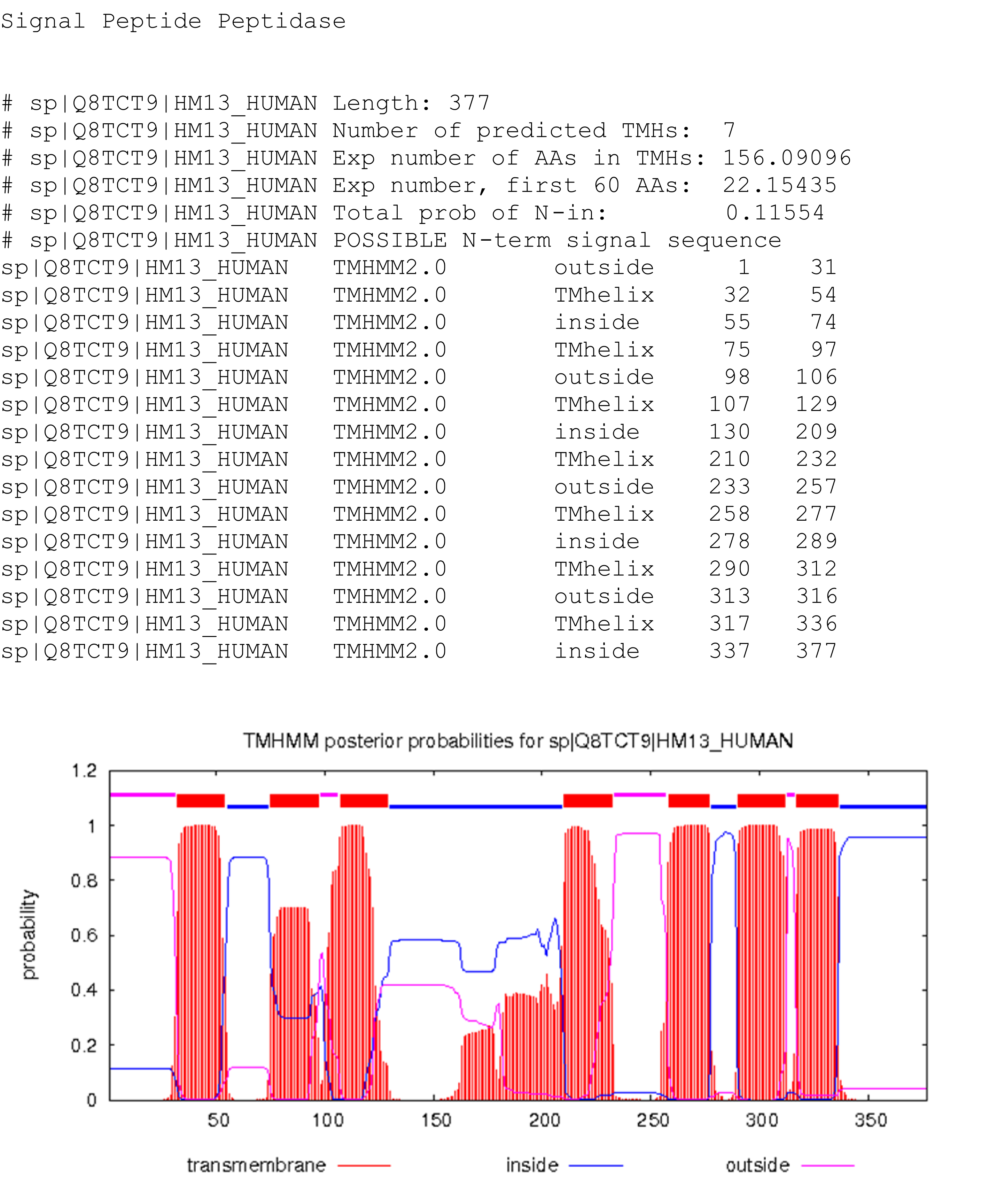

Supplement: S8 File — (TIFF) [file pone.0188344.s008.tiff]
